# Circular RNAs as Competing Endogenous RNAs in Cardiovascular and Cerebrovascular Diseases: Molecular Mechanisms and Clinical Implications

**DOI:** 10.3389/fcvm.2021.682357

**Published:** 2021-07-07

**Authors:** Xue Min, Dong-liang Liu, Xing-dong Xiong

**Affiliations:** ^1^Guangdong Provincial Key Laboratory of Medical Molecular Diagnostics, Institute of Aging Research, Guangdong Medical University, Dongguan, China; ^2^Institute of Biochemistry and Molecular Biology, Guangdong Medical University, Zhanjiang, China

**Keywords:** circRNA, ceRNA, cardiovascular and cerebrovascular diseases, molecular mechanism, clinical implication

## Abstract

Circular RNAs (circRNAs) represent a novel class of widespread and diverse endogenous RNA molecules. This unusual class of RNA species is generated by a back-splicing event of exons or introns, resulting in a covalently closed circRNA molecule. Accumulating evidence indicates that circRNA plays an important role in the biological functions of a network of competing endogenous RNA (ceRNA). CircRNAs can competitively bind to miRNAs and abolish the suppressive effect of miRNAs on target RNAs, thus regulating gene expression at the posttranscriptional level. The role of circRNAs as ceRNAs in the pathogenesis of cardiovascular and cerebrovascular diseases (CVDs) has been recently reported and highlighted. Understanding the underlying molecular mechanism could aid the discovery of therapeutic targets or strategies against CVDs. Here, we review the progress in studying the role of circRNAs as ceRNAs in CVDs, with emphasis on the molecular mechanism, and discuss future directions and possible clinical implications.

## Introduction

Cardiovascular and cerebrovascular diseases (CVDs) are general terms used to refer to all cardiac and cerebral diseases related to vasculopathy. Despite improvements in pharmacotherapy and surgical interventions, as well as lifestyle modifications, morbidity and mortality in patients with CVDs remain high in recent years (1). CVDs are still the leading cause of death worldwide (1). Current treatments primarily alleviate symptoms or slow down disease progression. To develop novel preventive and therapeutic strategies, we should elucidate and understand the underlying molecular mechanisms of these diseases.

Circular RNAs (circRNAs) were first discovered in plant viroids more than 40 years ago (2). A few years later, circRNAs were observed by electron microscopy in the cytoplasmic fractions of eukaryotic cells (3). However, they were mainly considered to be errors of the normal splicing process and did not receive much attention (4). In recent years, advances in the high-throughput sequencing technology and circRNA-specific bioinformatics algorithms have resulted in the discovery and identification of thousands of circRNAs (5–9). Indeed, circRNAs are diverse, abundant, and expressed in a tissue- and developmental stage-specific manner (5, 8). Although the functions of most circRNAs remain elusive, a select number of circRNAs are known to function as competitive endogenous RNAs (ceRNAs) by decoying miRNAs from other target transcripts, thereby controlling gene expression at the posttranscriptional level (10).

Recent studies have identified circRNAs as ceRNAs in many diseases including CVDs (11–13). Here, we reviewed the role of circRNAs as ceRNAs in atherosclerosis, myocardial infarction, cardiac fibrosis, heart failure, aneurysm, and stroke, with a special focus on the molecular mechanism, which provides a new direction for further research on the pathogenesis and treatment of CVDs.

## CircRNA SERVES AS ceRNA

Several years before the discovery of ceRNAs, or natural miRNA sponges, many studies had found that artificial miRNA sponges were able to specifically and effectively inhibit miRNA activity (14–16). These synthetic miRNA sponges are usually expressed from strong promoters, engineered to carry multiple binding sites for a miRNA or miRNA family of interest and have been shown to derepress miRNA targets both *in vitro* and *in vivo* (14). The sponging constructs are not only invaluable tools for miRNA loss-of-function studies *in vitro* and *in vivo*, but also critical for the development of RNA-based therapeutic applications (15, 16).

The theoretical and practical development of the artificial miRNA sponge laid an important foundation for the ceRNA hypothesis. In 2011, Salmena et al. postulated that all endogenous RNA transcripts sharing common miRNA response elements (MREs) can communicate with and regulate each other through competition for a limited pool of miRNAs (17). This hypothesis not only attributes new functions for non-coding RNA transcripts, but also implies coding-independent functions of protein-coding messenger RNAs (17). Given that any transcripts containing MREs can theoretically serve as ceRNAs, they may represent a widespread form of posttranscriptional gene regulation in a variety of physiological and pathophysiological processes.

**Figure 1:**
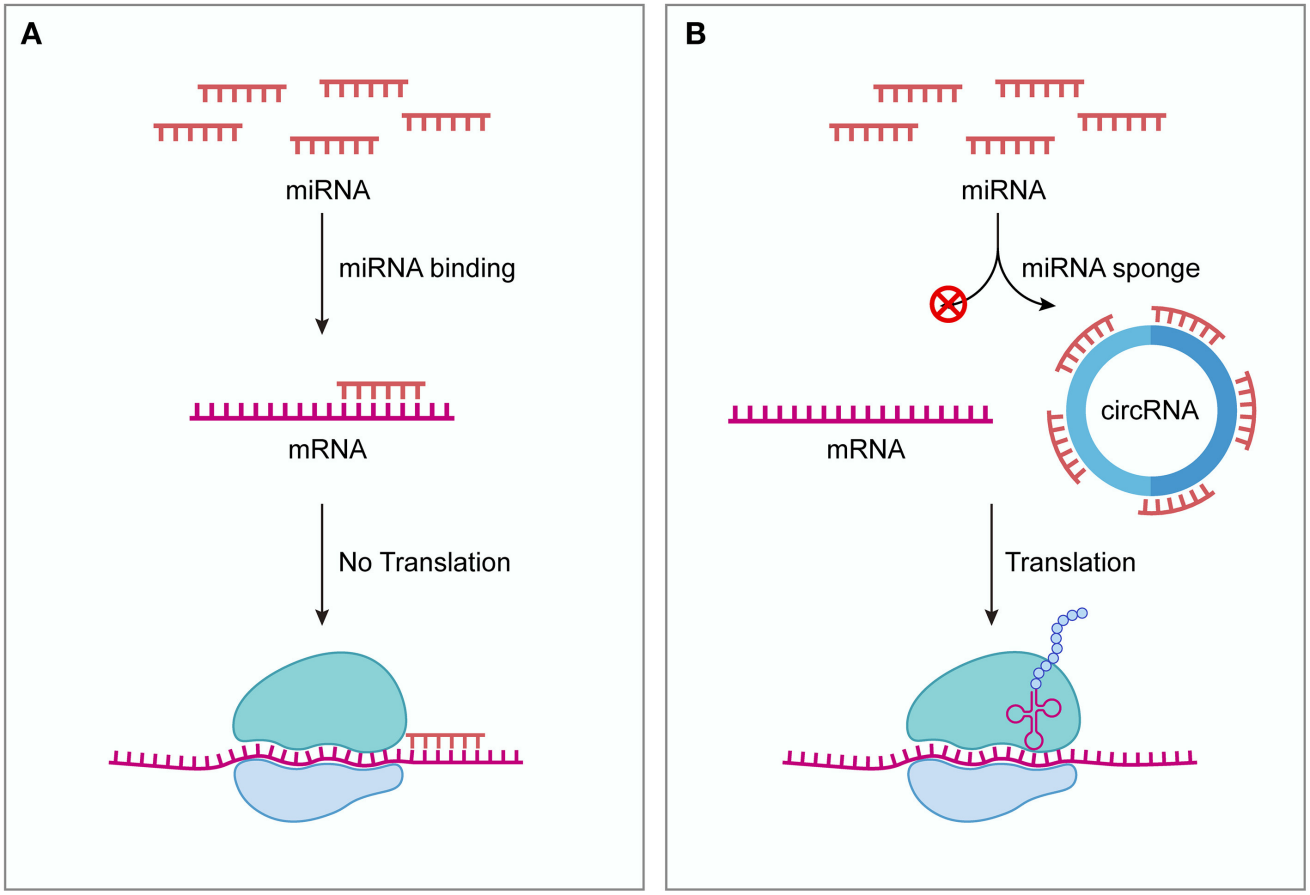
circRNAs function as ceRNAs. **(A)** Without competing transcripts circRNAs, miRNAs bind to target mRNAs through sequence complementarity, blocking the translation of target mRNAs. **(B)** circRNAs can competitively sequestrate miRNAs and derepress their target mRNAs, thus promoting the expression of target genes. ceRNA, competing endogenous RNA; circRNA, circular RNA.

In 2013, two independent groups firstly discovered that circRNAs can serve as ceRNAs (18, 19). Hansen et al. showed that ciRS-7 (also known as CDR1as) contains 73 conserved binding sites for miR-7 (19). ciRS-7 strongly binds to miR-7 as well as the miRNA effector Argonaute protein 2 (AGO2) and efficiently suppresses miR-7 activity, thereby attenuating the availability of miR-7 to bind to its target mRNAs. *In situ* hybridization showed that miR-7 and ciRS-7 displayed clear co-expression in mouse brain sections and in primary cells isolated from mouse brain, suggesting a high level of endogenous interactivity between them. Another research group demonstrated that ectopic expression of human ciRS-7 in zebrafish impairs midbrain development, consistent with the effect of miR-7 inhibition induced by morpholinos injection (18). The midbrain reduction induced by ciRS-7 expression in zebrafish could be partially rescued by expressing miR-7, indicating the interaction between ciRS-7 and miR-7. These two studies also serve as the first functional analysis of naturally expressed circRNAs. Acting as ceRNAs, circRNAs can bind to miRNAs and inhibit the function of miRNAs, thereby upregulating miRNA target gene expression (Figure 1). Because of the lack of free ends, circRNAs are more resistant to exonucleases and more stable than linear RNAs (18, 19). These natures of circRNAs are thought to facilitate their effectiveness as ceRNAs. Indeed, so far many circRNAs have been found to act as ceRNAs both under normal physiological conditions and diseased conditions (20).

## CircRNA AS ceRNA IN CVDs

### Atherosclerosis

Atherosclerosis (AS) is one group of the most common vasculopathies and a common pathological basis for many cardiovascular diseases, characterized by abnormal lipid metabolism and inflammatory reactions. A number of cells are involved in the pathology and pathophysiology of AS, including vascular endothelial cells (VECs) and vascular smooth muscle cells (VSMCs). Recent studies have found that circRNAs can effectively regulate the proliferation and migration of VECs and VSMCs by acting as ceRNAs, thus modulating the progression of AS (21, 22).

VECs comprise the inner layer of blood vessels and play crucial roles in vascular homeostasis. Endothelial cell injury and dysfunction are critical events that trigger the development of atherosclerotic lesions. Importantly, ox-LDL has been identified to contribute to the progression of AS by inducing VEC injury and apoptosis (23, 24). Zhang et al. found that the circRNA circ_0003204, which is upregulated in ox-LDL-induced human aortic endothelial cells (HAECs), can promote the expression of TGFβR2 and its downstream phospho*-*SMAD3 by sponging miR-370, leading to inhibition of the proliferation, migration, and capillary-like formation of HAECs exposed to ox-LDL (21). Another study revealed that circ_0124644 exacerbates ox-LDL-induced HUVECs injury through the miR-149-5p/PAPP-A axis (25). In addition, circ-USP36 can bind miR-98-5p, which results in increased levels of VCAM1, thus accelerating ox-LDL-induced apoptosis, inflammatory and viability inhibition of HUVEC cells (26).

VSMCs reside in the tunica media and maintain vascular tension. Abnormal proliferation of VSMCs accelerates the progression of atherosclerosis. A previous study reported that circACTA2 competitively binds miR-548f-5p and upregulates smooth muscle α-actin (α-SMA) expression, thereby facilitating stress fiber formation and cell contraction in human arterial smooth muscle cells (HASMCs) (27). α-SMA participates in the formation of filaments that are major components of the cytoskeleton which is essential for VSMC proliferation and migration (27). Another study reported that circ_Lrp6 competes endogenously by binding to miR-145, thus regulating VSMC migration, proliferation, and differentiation (22). More recently, it has been reported that CircMap3k5 functions as a miRNA sponge to decrease miR-22-3p repression of TET2 (13). As a consequence, CircMap3k5 inhibits the proliferation of smooth muscle cells (SMCs) and attenuates intimal hyperplasia (13). ox-LDL can promote the growth, migration, and differentiation of VSMCs, leading to VSMC dysfunction (28). Studies have shown that hsa_circ_0029589 and circ_0010283 are up-regulated in ox-LDL-induced VSMCs (29–32). Knockdown of these circRNAs ameliorates the dysfunction of VSMCs induced by ox-LDL and they exert these biological functions by serving as ceRNAs. VSMCs may preserve phenotype alterations from a highly quiescent and differentiated phenotype (contractile phenotype) to a proliferative and dedifferentiated phenotype (secretory phenotype) (33). The VSMC phenotypic switching is an early event in AS. circ_RUSC2, circ-SATB2, circDHCR24 and circ-Sirt1 have been found to regulate the phenotypic transformation of VSMCs by functioning as ceRNAs (11, 34–36). These circRNAs may be targets for regulating VSMC phenotypic modulation and serve as potential diagnostic biomarkers and therapeutic targets for AS.

### Myocardial Infarction

Myocardial infarction (MI), also called acute MI, is characterized by cardiac injury and remodeling caused by a sudden loss of oxygen supply due to acute coronary artery occlusion. Apoptosis has shown great contribution to cardiomyocyte loss during acute MI. Several studies have shown that circRNAs are involved in the regulation of cardiomyocyte apoptosis (37–41). The mitochondrial fission and apoptosis-related circRNA (MFACR) can mediate cardiomyocyte apoptosis and MI by exerting an endogenous competitive effect and regulating the expression of MTP18 (37). circNCX1 is increased in response to ROS and promotes the expression of the pro-apoptosis factor CDIP1 by targeting miR-133a-3p, eventually leading to the death of cardiomyocytes (38). Cdr1as is upregulated in MI mice, and promotes myocardial cell apoptosis and increases cardiac infarct size by targeting the Cdr1as/miR-7a axis (39). Conversely, circMACF1 can attenuate cardiomyocyte apoptosis through the miR-500b-5p-EMP1 axis (40). circ-Ttc3, as a miR-15b-5p sponge, promotes the expression of Arl2 and plays a cardioprotective role in MI (41).

The stimulation of endogenous cardiac regeneration is a promising approach to replace the lost myocardium after MI. Cardiomyocyte proliferation and angiogenesis are essential for structural and functional repair during cardiac regeneration. Gao et al. demonstrated that circFASTKD1 binds directly to miR-106a and relieve its inhibition of LATS1 and LATS2, thereby suppressing the YAP signaling pathway (42). The downregulation of circFASTKD1 ameliorates MI by promoting angiogenesis through the miR-106a-LATS1/2-YAP axis (42). Another study showed that circHipk3 acts as a sponge for miR-133a to promote CTGF expression (43). Upregulation of circHipk3 induces cardiac regeneration and angiogenesis, and decreases the infarct size after MI (43). Huang et al. clarified that circNfix can sponge miR-214 to promote Gsk3β expression and that the knockdown of circNfix promotes cardiomyocyte proliferation and angiogenesis after MI, attenuating cardiac dysfunction and improving prognosis (44).

### Cardiac Fibrosis

Cardiac fibrosis is defined as the replacement of normal myocardial tissues with non-beating fibrous tissues caused by the hyperfunction of fibroblasts and differentiation into myofibroblasts. It is a common pathological feature of the most adverse ventricular remodeling, such as MI and diabetic cardiomyopathy. Some circRNAs have been reported to exert pro-fibrosis effect in cardiac fibroblasts (CFs). circRNA_010567 and circRNA_000203 were upregulated in diabetic mouse myocardium and Ang-II-induced CFs (45, 46). circRNA_010567 can competitively bind to miR-141 and remove the inhibition of TGF-β1 by miR-141, thus increasing TGF-β1 levels (45). circRNA_000203 can competitively bind to anti-fibrotic miR-26b-5p, enhancing the fibrotic phenotype in CFs (46). Similarly, circPAN3 exhibits pro-fibrotic effects via the miR-221/FoxO3/ATG7 axis (47). Another study showed that circHIPK3 is markedly upregulated in CFs and heart tissues treated with Ang-II (48). Knockdown of circHIPK3 prevents Ang-II-induced cardiac fibrosis and improves diastolic function by sponging miR-29b-3p (48). Several circRNAs have been shown to exert anti-fibrotic effect in CFs. circNFIB, which is decreased in post-MI heart samples of mice and TGF-β-treated CFs, can directly sponge miR-433 to inhibit fibroblast proliferation (49). circ_LAS1L can inhibit the activation, proliferation, and migration of CFs by targeting the miR-125b-SFRP5 signaling axis (50). Herein, a better understanding of the molecular mechanisms that regulate cardiac fibrosis could aid the discovery of novel therapeutic approaches for the development of cardiac remodeling.

### Heart Failure

Cardiac hypertrophy is characterized by the enlargement of cardiomyocytes and thickening of the ventricular walls. It initially develops as an adaptive response to diverse patho-physiological stimuli; however, chronic sustained cardiac hypertrophy may eventually progress to heart failure (HF). Several reports have revealed that circRNAs function as ceRNAs in the development and progression of HF. Wang et al. found that overexpression of heart-related circRNA (HRCR) increases the expression of ARC by inhibiting miR-223 activity, which attenuates the development of cardiac hypertrophy and HF (51). Lim et al. found that circSlc8a1 inhibition could attenuate cardiac hypertrophy and HF by sponging miR-133a (52). It has been reported that circRNA_000203 exacerbates cardiac hypertrophy by competitively sponging miR-26b-5p and miR-140-3p to derepress Gata4, a pro-hypertrophic transcription factor (53). A recent study showed that circHIPK3 is upregulated in pressure-overload induced cardiomyocytes (54). Knockdown of circHIPK3 plays a protective role against cardiac hypertrophy by sponging miR-185-3p (54). These circRNAs hold promise as prospective therapeutic targets for HF.

### Aneurysm

Aneurysm is defined as a pathological condition featured by permanent localized dilation of the vessel wall, involving all layers. Growth impediment and apoptosis of VSMCs can contribute to aneurysm development. Recently, interactions between circRNAs and miRNAs have also been demonstrated in aneurysms. For example, hsa_circRNA_101238 can sequester miR-320a, increasing the expression of MMP9 and leading to thoracic aortic dissection (55). circ_0020397 plays an important role in the phenotypic modulation of intracranial aneurysms by promoting VSMC viability via the miR-502-5p/GREM1 axis (56). Additionally, CDR1as serves as a sponge for miR-7, which increases the expression of CKAP4 to facilitate the proliferation and suppress the apoptosis of VSMCs, leading to VSMC remodeling and progression of abdominal aortic aneurysm (AAA) (57). circCBFB facilitates VSMC proliferation and inhibits VSMC apoptosis via the circCBFB/miR-28-5p/GRIA4/LYPD3 axis (58). circCCDC66 sponges miR-342-3p to upregulate its host gene *CCDC66* in VSMCs and regulates VSMC apoptosis and proliferation (59). These newly identified mechanisms may provide novel options for the treatment of aneurysm.

### Stroke

Stroke is a common cardiovascular and cerebrovascular disease and one of the major causes of death and disability worldwide. During the hours after an ischemic stroke, neurons become permanently damaged and undergo cell death. Therefore, novel therapeutic approaches to rescue damaged neurons are urgently needed. The latest study conducted by Dai et al. revealed that knockdown of circHECTD1 attenuates neuronal injury caused by cerebral ischemia via the miR-133b/ TRAF3 axis (60). Chen et al. revealed that circUCK2 functions as a miR-125b-5p sponge to inhibit miR-125b-5p activity, resulting in an increase in GDF11 expression and a subsequent amelioration of neuronal injury (61). Wu et al. demonstrated that circTLK1 acts as an endogenous miR-335-3p sponge to increase the expression of TIPARP, resulting in the exacerbation of neuronal injury (62). It has been shown that circSHOC2 in ischemic-preconditioned astrocyte-derived exosomes attenuates ischemia-induced neuronal apoptosis and ameliorates neuronal damage by acting on the miR-7670-3p/SIRT1 axis (63). The above circRNAs may serve as potential targets for inducing neuroprotection against ischemic stroke.

Astrocytes are the most abundant cells in the brain and play important roles in maintaining normal brain function. Studies have demonstrated that treatments capable of decreasing infarct size are often accompanied by attenuated astrocyte responses (64, 65). Han et al. found that circHECTD1 acts as an endogenous miR-142 sponge to increase the expression of TIPARP, resulting in astrocyte activation, and thus contributes to cerebral infarction (66). Specific blockage of circHECTD1 may be a potential therapeutic target for the inhibition of astrocyte activation in stroke patients.

In addition, Bai et al. firstly reported that endothelial-mesenchymal transition (EndoMT) contributes to blood-brain barrier (BBB) damage during stroke pathogenesis (67). circDLGAP4 competitively binds miR-143 and remove the inhibition of HECTD1 by miR-143, thus increasing HECTD1 levels (67). Overexpression of circDLGAP4 significantly attenuates neuronal deficits, decreases infarct areas, and ameliorates BBB damage through the inhibition of EndoMT (67).

## Methods for Characterization of circRNA-miRNA Interactions

To investigate the interaction between circRNAs and miRNAs, several bioinformatic tools can be used. For instance, different miRNA-target prediction algorithms, including TargetScan (68), miRanda (69), and PITA (70) have greatly facilitated the discovery of circRNA-miRNA interactions. In addition, some tools incorporate gene expression data including AGO crosslinking-immunprecipitation (CLIP) data, which improve the capability to predict physiologically relevant circRNA-miRNA interactions. starBase 2.0 (http://starbase.sysu.edu.cn/) is the first database containing circRNA-miRNA interactions based on Ago and RNA binding protein (RBP) binding sites (71). It provides comprehensive interaction networks of ncRNAs, mRNA and RBP from 108 CLIP-Seq datasets. CircInteractome (http://circinteractome.nia.nih.gov/) combines many features from other websites, such as circBase, TargetScan, StareBase (72). Similarly, it can be used to explore the interacting miRNAs of circRNAs. Since every database uses distinct rules of targeting and produces different prediction outcomes, a combination of various databases may provide information in a reliable way.

In addition to the above mentioned prediction methods, experimental validation is required to prove that a circRNA has a bona fide miRNA sponging function. First, dual-luciferase reporter assays can be utilized to screen the potential interacting miRNAs. The circRNA sequences containing wild-type (WT) or mutated miRNA binding sites are inserted into luciferase reporter vectors. Transfection of a miRNA mimic can reduce the luciferase activities in the WT group, but not in the mutated group. Second, RNA fluorescence *in situ* hybridization (RNA-FISH) can be used to assess the colocalization of circRNAs with miRNAs in cells and tissues. RNA-FISH probe should target circRNA backspliced junctions in order to avoid recognition of their cognate linear RNAs. It is important to note that RNA-FISH signals for circRNAs and miRNAs appear as distinct puncta in the cell, not as the diffuse signals typically seen when detecting protein. Third, AGO2 RNA immunoprecipitation (RIP) can be employed to confirm a physical interaction between the circRNA, miRNA and AGO2. It is a more direct evidence supporting the miRNA sponging role of a circRNA. Fourth, RNA pull-down assay can be performed using biotinylated probes designed specifically for the circRNA/miRNA of interest. After confirming that the circRNA/miRNA is successfully enriched in the pull-down material, the interacting miRNA/circRNA molecules can be studied by RT-qPCR analysis.

## Conclusion And Future Prospects

As discussed in this review, many circRNAs serve as ceRNAs to mediate miRNA functions in CVDs (Table 1). These circRNA-miRNA-mRNA competitive endogenous RNA regulatory networks represent a novel section in the pathogenesis and development of CVDs. As lots of circRNAs are deregulated or show a disease-specific profile in CVDs and might play a role in the progression of CVDs, they might serve as promising biomarkers for early diagnosis or therapeutic targets in the future. However, how to effectively regulate circRNA levels in target cells is now still unresolved. Extracellular vesicles are naturally occurring RNA carriers that can induce physiological changes in target cells through the transfer of numerous molecules. These vesicles can be adapted for the delivery of circRNAs or siRNAs targeting the specific backspliced sequence of circRNA. Therefore, exogenous upregulation or downregulation of circRNAs to regulate miRNAs could become a novel therapy for CVD treatment. In addition, a recent study found that artificial circRNA sponges can competitively inhibit pro-hypertrophic miR-132 and−212, thereby attenuating pressure overload-induced cardiac hypertrophy (73). Thus, introducing synthetic circRNAs sponges into target cells might also serve as new efficient approaches for future CVD therapy. Indeed, the superior ability of circRNA in sponging miRNAs and its unique cellular stability confer them great potential for use as therapeutic drugs. Nevertheless, the real clinical application of circRNAs as molecular drugs or targets is challenging. How to ensure therapeutic efficacy and safety, and avoid off-target adverse effects awaits additional and extensive investigation.

**Table 1:** CircRNAs as ceRNAs in CVDs.

**Diseases**	**circRNAs**	**miRNAs**	**mRNAs**	**Functions**	**Number of miRNA binding sites**	**References**
Atherosclerosis	circ_0003204	miR-370	TGFβR2	Inhibited proliferation, migration and tube formation of HAECs exposed to ox-LDL	2	(21)
	circ_0124644	miR-149-5p	PAPP-A	Intensified the ox-LDL-induced HUVECs injury	1	(25)
	circUSP36	miR-98-5p	VCAM1	Accelerated ox-LDL-induced apoptosis, inflammatory and viability inhibition of HUVEC cells	1	(26)
	circACTA2	miR-548f-5p	α-SMA	Facilitated stress fiber formation and cell contraction in HASMCs	1	(27)
	circLrp6	miR-145	FASCIN, Yes1, and Lox	Promoted VSMC migration, proliferation, and differentiation	7	(22)
	circMAP3K5	miR-22-3p	TET2	Inhibited SMC proliferation and attenuated intimal hyperplasia	1	(13)
	circ_0029589	miR-214-3p	STIM1	Regulated VSMC proliferation, migration and invasion	1	(29)
	circ_0029589	miR-370	FOXO1	Promoted VSMC the proliferation and migration ability	1	(30)
	circ_0029589	miR-214-3p	Wnt3	Promoted cell growth, migration and inflammation in VSMCs	1	(31)
	circ_0010283	miR-133a-3p	PAPPA	Promoted ox-LDL-induced proliferation, migration and invasion in HVSMCs	1	(32)
	circRUSC2	miR-661	SYK	Promoted VSMC proliferation, enhanced cell migration and inhibited cell apoptosis	–	(34)
	circSATB2	miR-939	STIM1	Promoted proliferation, migration and inhibited apoptosis of VSMC	–	(35)
	circDHCR24	miR-149-5p	MMP9	Promoted HA-VSMC proliferation, migration and phenotypic switching	1	(36)
	circSirt1	miR-132*/*212	SIRT1	Inhibited inflammatory phenotypic switching of VSMCs	3	(11)
Myocardial infarction	circMFACR	miR-652-3p	MTP18	Regulated mitochondrial dynamics and apoptosis in heart	15	(37)
	circNCX1	miR-133a-3p	CDIP1	Promoted cardiomyocyte apoptosis	8	(38)
	circCdr1as	miR-7a	PARP, SP1	Promoted MCM cell apoptosis	–	(39)
	circMACF1	miR-500b-5p	EMP1	Attenuated cardiomyocyte apoptosis	1	(40)
	circTtc3	miR-15b	Arl2	Protected cardiomyocyte from apoptosis	1	(41)
	circFASTKD1	miR-106a	LATS1, LATS2	Suppressed endothelial cell growth, migration, mobility and angiogenesis.	1	(42)
	circHipk3	miR-133a	CTGF	Promoted coronary vessel endothelial cell proliferation, migration, tube-forming capacity and angiogenesis	2	(43)
	circNfix	miR-214	Gsk3β	Inhibited neonatal cardiomyocyte proliferation and angiogenesis	3	(44)
Cardiac fibrosis	circ_010567	miR-141	TGF-β1	Regulated myocardial fibrosis	1	(45)
	circ_000203	miR-26b-5p	Col1a2, CTGF	Increased cardiac fibrosis	2	(46)
	circPAN3	miR-221	FoxO3	Exerted profibrotic role	1	(47)
	circHIPK3	miR-29b-3p	a-SMA, COL1A1, COL3A1	Promoted cardiac fibroblast proliferation and migration	2	(48)
	circNFIB	miR-433	AZIN1, JNK1	Inhibited cardiac fibroblast activation, proliferation and migration	1	(49)
	circLAS1L	miR-125b	SFRP5	Inhibited cardiac fibroblast activation, proliferation, migration and promoted apoptosis	1	(50)
Heart failure	circHRCR	miR-223	ARC	Attenuated the development of cardiac hypertrophy and heart failure	6	(51)
	circSlc8a1	miR-133a	Srf, Ctgf, Adrb1, Adcy6	Induced heart failure	10	(52)
	circ_000203	miR-26b-5p miR-140-3p	Gata4 Gata4	Aggravated cardiac hypertrophy and impaired heart function	1 1	(53)
	circHIPK3	miR-185-3p	CASR	Regulated cardiac hypertrophy	1	(54)
Aneurysm	circ_101238	miR-320a	MMP9	Involved in the pathogenesis of TAD	1	(55)
	circ_0020397 circCDR1as	miR-502-5p miR-7	GREM1 CKAP4	Promoted VSMC viability Facilitated the proliferation and suppressed the apoptosis of VSMCs	1 1	(56) (57)
	circCBFB	miR-28-5p	LYPD3, GRIA4	Exerted anti-apoptosis effects in VSMCs	1	(58)
	circCCDC66	miR-342-3p	CCDC66	Regulated VSMC apoptosis and proliferation	1	(59)
Stroke	circHECTD1	miR-133b	TRAF3	Attenuated the inhibition of OGD-caused cell apoptosis and NF-κB activation in HT22 cells	1	(60)
	circUCK2	miR-125b-5p	GDF11	Reduced neuronal apoptosis	–	(61)
	circTLK1	miR-335-3p	TIPARP	Aggravated neuronal injury	1	(62)
	circSHOC2	miR-7670-3p	SIRT1	Suppressed neuronal apoptosis and ameliorated neuronal damage	4	(63)
	circHECTD1	miR-142	TIPARP	Promoted astrocyte autophagy	1	(66)
	circDLGAP4	miR-143	HECTD1	Attenuated neuronal deficits, decreased infarct areas, and ameliorated BBB damage	1	(67)

–*, Not available*.

Despite growing evidence for the phenotypic effects of circRNA deregulation in CVDs, the current ceRNA hypothesis is still controversial regarding whether they are really active in the endogenous cellular context. Importantly, optimal ceRNA-mediated regulation is observed when miRNA and ceRNA levels are near equimolarity. The levels of circRNA need to be high enough to relieve the miRNA repression on mRNA. Thus, methods to quantitatively determine absolute expression levels of circRNA and miRNA will allow a more precise determination of ceRNA effectiveness of circRNAs in diverse physiological and pathological conditions. Another important factor is the number of miRNA-binding sites. CircRNAs that harbor multiple binding sites for the same miRNAs may serve as better ceRNA candidates than those with fewer sites. Among the circRNAs we discussed in this review, with the exception of MFACR (37), circSlc8a1 (52), circNCX1 (38), circLrp6 (22), HRCR (51), which contains 15, 10, 8, 7, 6 binding sites for miR-652-3p, miR-133a, miR-133a-3p, miR-145, miR-223, respectively, the majority of circRNAs harbor only a single or very few binding sites for the same miRNAs (Table 1). To function as efficient miRNA sponges, the copy numbers of these circRNAs that contain minimal numbers of miRNA-binding sites should attain levels comparable to the miRNAs. However, most of the researches are lack of assessments of absolute levels of circRNAs and miRNAs. Therefore, assessing the stoichiometry of a circRNA with the interacting miRNA is clearly needed in future research. In addition, factors such as miRNA/circRNA binding affinity and RBPs that could occupy MREs can also affect the ceRNA activity of circRNAs. The binding affinity of miRNA and its RNA target, to a great extent, is influenced by matching between MREs and seed sequences. In general, 8-mer seed match has higher affinity than those with 7-mer, and 6-mer has weakest miRNA target-site efficacy (74). As a competitor, circRNA should have a better affinity to miRNA than target mRNAs. RBP can interfere with circRNA-miRNA interactions by directly occupying RNA target sites, or by altering the circRNA secondary structure to influence its affinity for miRNAs. Therefore, technologies such as HITS-CLIP/iCLIP/PAR-CLIP, the mass-spectrometry based quantitative proteomic approaches for capturing RNA-interactome and measuring circRNA-binding protein abundance will help to assess the effect of competitive binding of RBP to MREs.

In summary, circRNAs acting as ceRNAs play important roles in CVDs. Research on the circRNA-mediated ceRNA regulatory network will open up new avenues for basic CVD research, as well as stimulate the development of novel diagnostic and therapeutic strategies for CVDs.

## Author Contributions

XM and D-lL: wrote the manuscript. X-dX: critically reviewed the manuscript. All authors contributed to the article and approved the submitted version.

## Conflict of Interest

The authors declare that the research was conducted in the absence of any commercial or financial relationships that could be construed as a potential conflict of interest.
